# Perioperative outcomes of total and unicompartmental knee arthroplasty in patients with Type 2 diabetes mellitus: A propensity score–matched analysis

**DOI:** 10.1002/jeo2.70917

**Published:** 2026-09-22

**Authors:** Yu Mori, Kunio Tarasawa, Ryuichi Kanabuchi, Hidetatsu Tanaka, Naoko Mori, Kiyohide Fushimi, Toshimi Aizawa, Kenji Fujimori

**Affiliations:** ^1^ Department of Orthopaedic Surgery Tohoku University Graduate School of Medicine Sendai Miyagi Japan; ^2^ Department of Health Administration and Policy Tohoku University Graduate School of Medicine Sendai Miyagi Japan; ^3^ Department of Radiology Akita University Graduate School of Medicine Akita Japan; ^4^ Department of Health Policy and Informatics Institute of Science Tokyo Tokyo Japan

**Keywords:** deep vein thrombosis, postoperative delirium, total knee arthroplasty, Type 2 diabetes mellitus, unicompartmental knee arthroplasty

## Abstract

**Purpose:**

Type 2 diabetes mellitus is a common comorbidity in patients undergoing knee arthroplasty and is associated with higher risks of thromboembolic, infectious and postoperative cognitive complications. The extent to which total knee arthroplasty (TKA) and unicompartmental knee arthroplasty (UKA) differ in short‐term perioperative risk among patients with Type 2 diabetes remains unclear.

**Methods:**

We performed a retrospective cohort study using Japan's nationwide Diagnosis Procedure Combination database, including patients with Type 2 diabetes who underwent primary TKA or UKA for osteoarthritis between April 2016 and March 2023. Outcomes included deep vein thrombosis, pulmonary embolism, surgical site infection, postoperative delirium, blood transfusion, length of hospital stay and discharge destination. A 1:1 propensity score (PS)–matched analysis was performed using demographic and clinical characteristics. Additional PS–matched and interaction analyses were performed in patients without Type 2 diabetes and in the overall cohort, respectively.

**Results:**

Among 263,838 eligible patients, 49,373 with Type 2 diabetes underwent TKA and 5,801 underwent UKA; 5783 matched pairs were analysed. After matching, TKA was associated with higher incidences of deep vein thrombosis (odds ratio [OR] 1.46; 95% confidence interval [CI] 1.29–1.66) and postoperative delirium (OR 2.35; 95% CI 1.39–3.96), longer hospital stay, lower rates of direct discharge to home and greater transfusion volume. Similar differences were observed in patients without Type 2 diabetes, and no significant interactions between diabetes status and surgical procedure were observed for deep vein thrombosis or postoperative delirium.

**Conclusions:**

Among patients with Type 2 diabetes undergoing knee arthroplasty, UKA was associated with a more favourable short‐term perioperative profile than TKA. Similar differences in deep vein thrombosis and postoperative delirium were observed in patients without Type 2 diabetes, with no significant interaction by diabetes status, suggesting that these differences were not specific to diabetes.

**Level of Evidence:**

Level III.

**Abbreviations**
CI: confidence interval
DPC: Diagnosis Procedure Combination
OR: odds ratio
SMD: standard mean difference
TKA: total knee arthroplasty
UKA: unicompartmental knee arthroplasty

## INTRODUCTION

Type 2 diabetes mellitus is a prototypical lifestyle‐related disease, and its global prevalence continues to increase, with many countries including Japan facing rising disease burden and the accumulation of long‐term complications [31, 38]. In the context of population aging and the growing number of total knee arthroplasty (TKA) and unicompartmental knee arthroplasty (UKA) procedures performed for osteoarthritis, Type 2 diabetes is a common and clinically important comorbidity among patients undergoing knee arthroplasty [5, 34, 45]. Chronic hyperglycaemia in patients with diabetes leads to progressive vascular damage, impaired immune function and musculoskeletal frailty, all of which have been implicated in the increased incidence of postoperative complications such as perioperative thromboembolic events and infections [2, 4]. In particular, venous thromboembolism, surgical site infection and reoperation or prolonged hospitalization due to delayed wound healing not only markedly compromise functional outcomes and quality of life, but also directly contribute to greater healthcare costs [7, 36, 42]. Therefore, accurate assessment and reduction of postoperative complication risk in patients with diabetes undergoing knee arthroplasty represent clinically important challenges.

Type 2 diabetes mellitus has consistently been identified as a comorbidity that adversely affects postoperative outcomes in patients undergoing TKA [35]. Existing studies have shown that patients with diabetes experience higher rates of periprosthetic joint infection, surgical site infection, venous thromboembolism, cardiovascular events, readmission and reoperation compared with those without diabetes and may have slower recovery of physical function and patient‐reported outcomes [1, 6, 29, 35]. In contrast, UKA is generally regarded as a less invasive procedure, with reports of shorter operative time and hospital stay, more rapid early recovery of range of motion and gait function and lower incidences of short‐term complications in carefully selected patient cohorts [15, 18, 20, 37]. However, most of these findings derive from populations that do not specifically focus on the presence of diabetes, and, to our knowledge, no large‐scale studies have directly compared postoperative complication profiles between TKA and UKA in patients with Type 2 diabetes [11, 12]. Consequently, it remains unclear which procedure offers a safer and more resource‐efficient strategy for the growing number of knee arthroplasty candidates living with diabetes.

The Japanese Diagnosis Procedure Combination (DPC) database is a nationwide case‐mix system that integrates detailed clinical and procedural information and has been widely used to investigate postoperative complications and outcomes after orthopaedic surgeries, including knee arthroplasty [8, 9, 13, 17, 23, 24, 26, 27, 28, 39, 43]. Given these gaps in the literature, large‐scale database studies with meticulous adjustment for patient characteristics and comorbidities are needed to clarify the true postoperative risk profile associated with different knee arthroplasty procedures in patients with Type 2 diabetes. Therefore, we used Japan's nationwide DPC database to identify patients with Type 2 diabetes who underwent TKA or UKA and to compare the incidence of in‐hospital postoperative complications between these two procedures after adjustment for demographic and clinical factors. We evaluated postoperative outcomes including venous thromboembolism, pneumonia, postoperative delirium, cerebrovascular events, surgical site infection, periprosthetic fracture, length of hospitalization, direct discharge to home and blood transfusion. To minimize confounding, comparisons were conducted between propensity score (PS)–matched cohorts based on age, sex, anaesthesia type, surgical procedure (TKA or UKA), Charlson Comorbidity Index (CCI) and relevant comorbidities. We hypothesized that, even among patients with Type 2 diabetes receiving contemporary standardized perioperative care, TKA would be associated with higher risks of thromboembolic and infectious complications than UKA.

## METHODS

### Study design

This study was conducted in accordance with the ethical principles of the Declaration of Helsinki and was approved by our Institutional Review Board. Data were retrospectively extracted from Japan's nationwide DPC database, as previously described [19, 21]. The study included patients with Type 2 diabetes mellitus who underwent knee arthroplasty between April 2016 and March 2023. Informed consent was obtained using an opt‐out approach. Upon admission, patients were informed that their clinical data could be used for academic research and were given the opportunity to decline participation. No personally identifiable information was included in the dataset. During the study period, approximately 1100 hospitals participating in Japan's DPC system consistently contributed medical records and approved their use for research.

Patients with Type 2 diabetes were identified using the International Statistical Classification of Diseases and Related Health Problems, 10th Revision (ICD‐10) code E11. Patients with Type 2 diabetes who underwent TKA or UKA at participating hospitals between April 2016 and March 2023 were included in the analysis. This nationwide cohort was used to compare perioperative outcomes between TKA and UKA in patients with Type 2 diabetes.

The primary postoperative outcomes included deep vein thrombosis, pulmonary embolism, surgical site infection, postoperative delirium, blood transfusion, length of hospital stay and discharge destination. Postoperative delirium was identified using the following ICD‐10 codes newly recorded during the index hospitalization after surgery: F050, F051, F058, F059, F010, F011, F012, F019, F03, F107, G238, G300, G301, G308, G309, G310 and G318 [23, 25]. Pre‐existing cognitive impairment, including dementia, was incorporated as a covariate in the PS model. Only ICD‐10 codes newly recorded during the index hospitalization after surgery were considered postoperative delirium; diagnoses recorded at admission were not classified as postoperative outcomes.

The primary indication for knee arthroplasty was osteoarthritis, identified using ICD‐10 codes M170–M175 and M179. Patients with rheumatoid arthritis (ICD‐10 codes M0586, M0606, M0686 and M0696) were excluded from the study. The use of antithrombotic agents was evaluated based on medications administered during the index hospitalization, as recorded in the administrative claims data. Information on chronic preoperative antithrombotic therapy before admission was not available. Therefore, antithrombotic medication use was analysed descriptively and was not included in the PS model.

Patients with Type 2 diabetes who underwent TKA or UKA were identified based on three key criteria: (1) the main diagnosis, (2) the primary reason for hospitalization and (3) the condition requiring the greatest medical resources. Patients who underwent bilateral simultaneous procedures were included, whereas those who underwent revision knee arthroplasty were excluded. Arthroplasty performed for trauma or bone tumours was also excluded from the analysis.

### PS matching

This study compared postoperative outcomes between patients with Type 2 diabetes mellitus who underwent TKA and those who underwent UKA. A 1:1 PS–matching analysis was performed to balance baseline characteristics between the two groups. The PS model included age, sex, body mass index, general anaesthesia, bilateral simultaneous procedures, hypertension, ischaemic heart disease, chronic kidney disease, hyperlipidemia, cerebrovascular disease, cognitive impairment and the CCI. The discriminatory ability of the PS model was evaluated using the C‐statistic. PSs were estimated using logistic regression and matched by nearest‐neighbour matching without replacement, with a caliper width of 0.2 times the standard deviation of the logit of the PS, as previously described [18]. This approach yielded a well‐balanced cohort of patients undergoing TKA and UKA, enabling robust comparisons of perioperative outcomes between the two surgical procedures.

### Statistical analysis

Data are presented as the mean ± standard deviation. Baseline characteristics were compared between patients who underwent TKA and those who underwent UKA using the standard mean difference (SMD), *χ* ^2^ test or Student's *t* test before and after PS matching. Perioperative outcomes were similarly compared between the unmatched and PS–matched cohorts.

Multivariable logistic regression analyses were subsequently performed in the unmatched cohort to identify independent risk factors for major postoperative complications that showed significant between‐group differences after PS matching. The models included clinically relevant covariates, including surgical procedure (TKA vs. UKA), age, sex, body mass index, bilateral simultaneous procedures, hypertension, ischaemic heart disease, chronic kidney disease, hyperlipidemia, cerebrovascular disease, cognitive impairment and the CCI.

As an additional analysis, patients without Type 2 diabetes mellitus who underwent TKA or UKA were identified, and one‐to‐one PS matching was performed using the same PS matching approach as that used for patients with Type 2 diabetes mellitus. Perioperative outcomes were compared between the PS–matched TKA and UKA groups. In addition, interaction analyses were performed in the overall cohort before PS matching to assess whether the presence of Type 2 diabetes mellitus modified the associations between surgical procedure and postoperative deep vein thrombosis or delirium. The interaction term between diabetes status and surgical procedure was added to the multivariable logistic regression models using the same covariates described above.

Given the large sample size, a more stringent threshold for statistical significance was applied. All statistical tests were two‐sided, and *p* values < 0.001 were considered statistically significant. Statistical analyses were performed using JMP Version 18 (SAS Institute).

## RESULTS

**Figure 1:**
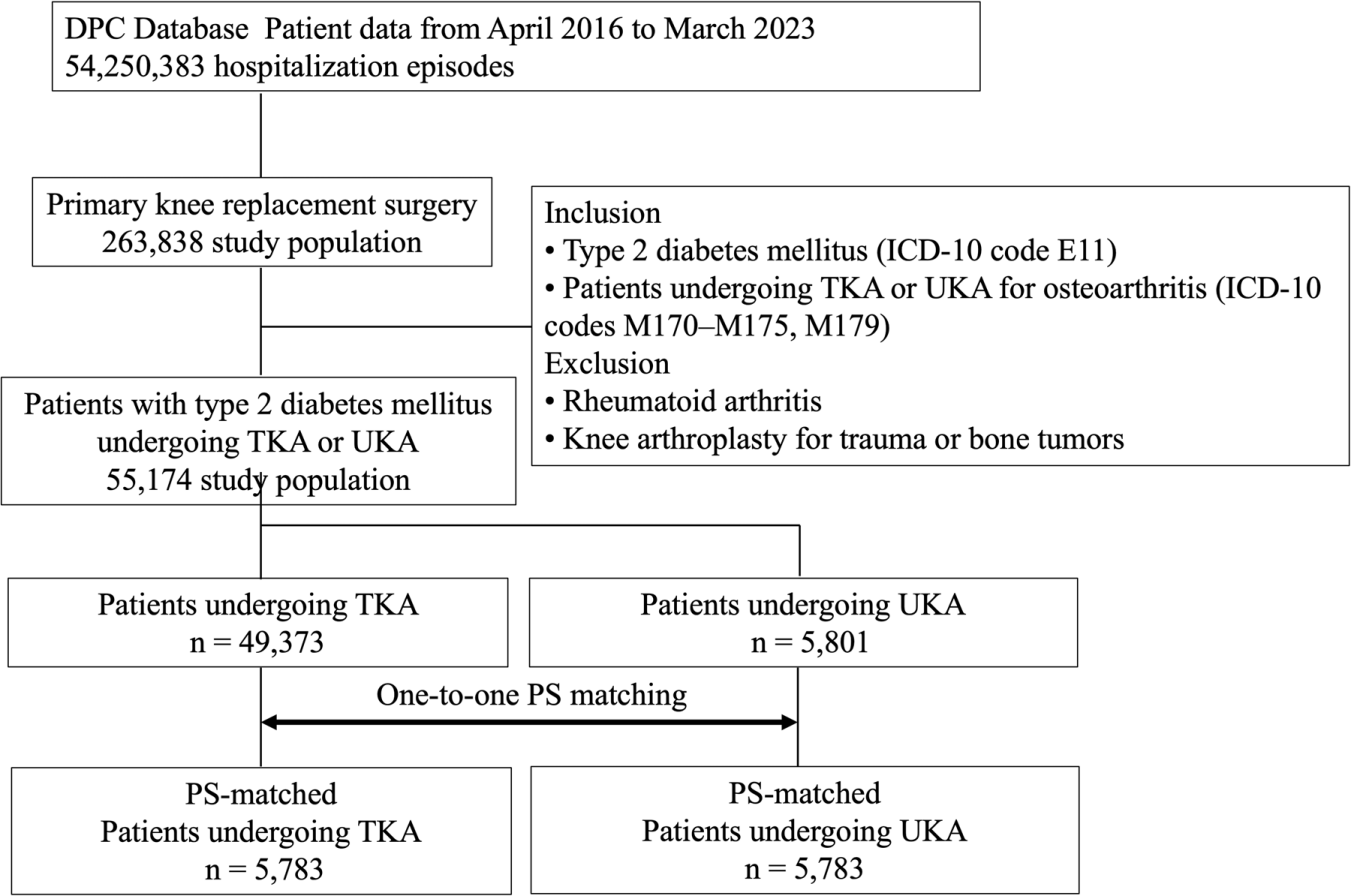
The flow diagram illustrates the selection of patients with Type 2 diabetes mellitus who underwent knee arthroplasty for osteoarthritis in the DPC database and the subsequent PS matching. It summarizes the inclusion and exclusion criteria used to identify eligible TKA and UKA cases and the steps employed to construct the 1:1 PS–matched cohorts. DPC, Diagnosis Procedure Combination; ICD‐10, International Statistical Classification of Diseases and Related Health Problems, 10th Revision; PS, propensity score; TKA, total knee arthroplasty; UKA, unicompartmental knee arthroplasty.

Figure 1 illustrates the patient selection flow. From the nationwide database covering April 2016 to March 2023, 263,838 patients met the predefined inclusion and exclusion criteria. Of these, 49,373 patients with Type 2 diabetes mellitus underwent TKA and 5801 underwent UKA. After 1:1 PS matching based on age, sex, body mass index, general anaesthesia, CCI, comorbid conditions and bilateral simultaneous procedures, 5783 matched pairs were included in the final comparative analysis.

Table 1 summarizes the baseline characteristics of patients with Type 2 diabetes who underwent TKA or UKA. Before PS matching, the TKA group included a higher proportion of women and had higher body mass index and CCI values than the UKA group, with SMDs exceeding 0.1. After 1:1 PS matching, all baseline covariates were well balanced, with SMDs below 0.1. The PS model demonstrated good discrimination, with a C‐statistic of 0.83.

**Table 1:** Characteristics of patients with Type 2 diabetes mellitus before and after PS matching.

	Before PS matching			After PS matching		
	TKA	UKA	SMD	TKA	UKA	SMD
*n*	49,373	5801		5783	5783	
Sex						
Men	11,594 (23.5%)	2012 (34.7%)	0.248	1907 (33.0%)	2006 (34.7%)	0.036
Woman	37,779 (76.5%)	3789 (65.3%)		3876 (67.0%)	3777 (65.3%)	
Age	74.5 ± 7.4	74.4 ± 7.3	0.008	74.5 ± 7.3	74.4 ± 7.3	0.008
BMI	27.2 ± 4.8	26.1 ± 3.8	0.256	26.1 ± 4.1	26.1 ± 3.8	0.015
CCI	1.55 ± 0.88	1.42 ± 0.79	0.141	1.41 ± 0.75	1.42 ± 0.79	0.017
Hypertension	20,121 (40.8%)	2157 (37.2%)	0.073	2045 (35.4%)	2150 (37.2%)	0.038
Ischaemic cardiac disease	3902 (7.9%)	442 (7.6%)	0.011	428 (7.4%)	441 (7.6%)	0.009
Chronic renal dysfunction	1288 (2.6%)	122 (2.1%)	0.034	118 (2.0%)	122 (2.1%)	0.005
Cerebrovascular disease	1915 (3.9%)	228 (3.9%)	0.003	214 (3.7%)	227 (3.9%)	0.012
Hyperlipidemia	11,069 (22.4%)	1313 (22.6%)	0.005	1274 (22.0%)	1309 (22.6%)	0.015
Cognitive impairment	664 (1.3%)	55 (1.0%)	0.038	48 (0.8%)	54 (0.9%)	0.011
General anaesthesia	45,226 (91.6%)	5173 (89.2%)	0.084	5132 (88.8%)	5158 (89.2%)	0.015
Bilateral surgery	3243 (6.6%)	233 (4.0%)	0.115	223 (3.9%)	233 (4.0%)	0.009

*Note*: One‐to‐one PS matching was performed. Data are shown as mean ± standard deviation. An SMD < 0.1 was considered indicative of adequate covariate balance.

Abbreviations: BMI, body mass index; CCI, Charlson Comorbidity Index; PS, propensity score; SMD, standard mean difference; TKA, total knee arthroplasty; UKA, unicompartmental knee arthroplasty.

Table 2 compares postoperative outcomes between patients with Type 2 diabetes undergoing TKA and UKA. Before PS matching, the TKA group had significantly higher incidences of deep vein thrombosis, postoperative delirium and surgical site infection than the UKA group. Patients undergoing TKA also had a longer hospital stay, a lower rate of discharge to home and a greater transfusion volume on the day of surgery.

**Table 2:** Comparison of complications before and after PS matching.

	Before PS matching			After PS matching			
	TKA (*n* = 49,373)	UKA (*n* = 5801)	*p* value	TKA (*n* = 5783)	UKA (*n* = 5783)	Odds ratio	*p* value
						95% CI	
Deep vein thrombosis	4477 (9.1%)	361 (6.2%)	<0.0001*	528 (9.1%)	361 (6.2%)	1.46 (1.29–1.66)	<0.0001*
Pulmonary embolism	156 (0.3%)	8 (0.1%)	0.018	18 (0.3%)	8 (0.1%)	2.25 (0.98–5.17)	0.05
Pneumonia	99 (0.2%)	4 (0.1%)	0.028	8 (0.1%)	4 (0.1%)	2.00 (0.60–6.63)	0.25
Delirium	372 (0.8%)	20 (0.3%)	0.0005*	47 (0.8%)	20 (0.4%)	2.35 (1.39–3.96)	0.0009*
Cerebrovascular event	264 (0.5%)	29 (0.5%)	0.73	26 (0.5%)	29 (0.5%)	0.67 (0.11–4.00)	0.66
Surgical site infection	623 (1.3%)	38 (0.7%)	<0.0001*	66 (1.1%)	38 (0.7%)	1.74 (1.17–2.58)	0.006
Periprosthetic fracture	28 (0.1%)	9 (0.2%)	0.006	3 (0.4%)	9 (0.1%)	0.33 (0.09–1.23)	0.08
Length of hospitalization (days)	29.2 ± 15.2	23.9 ± 12.9	<0.0001*	28.6 ± 13.8	23.9 ± 12.8		<0.0001*
Discharge to home	40,489 (82.0%)	5280 (91.0%)	<0.0001*	4760 (82.3%)	5265 (91.0%)	0.94 (0.89–0.92)	<0.0001*
Blood transfusion Day 0 (unit)	0.06 ± 0.41	0.01 ± 0.12	<0.0001*	0.06 ± 0.44	0.01 ± 0.11		<0.0001*

*Note*: One‐to‐one PS matching was performed. Odds ratios are expressed for TKA relative to UKA (reference).

Abbreviations: CI, confidence interval; PS, propensity score; TKA, total knee arthroplasty; UKA, unicompartmental knee arthroplasty.

* *p* values of <0.001 are considered significant by the *χ* ^2^ test and Student's *t* test.

After PS matching, the incidences of deep vein thrombosis and postoperative delirium remained significantly higher in the TKA group, whereas the difference in surgical site infection was no longer statistically significant. The TKA group also continued to have a longer hospital stay, a lower rate of discharge to home and a greater transfusion volume on the day of surgery than the UKA group. No significant between‐group differences were observed in other postoperative complications, including pulmonary embolism and cerebrovascular events.

Univariate logistic regression analysis demonstrated that TKA was significantly associated with an increased risk of deep vein thrombosis (odds ratio [OR], 1.46; 95% confidence interval [CI], 1.29–1.66; *p* < 0.0001), corresponding to an absolute risk difference of 2.8% (95% CI, 1.9–3.9). TKA was also significantly associated with postoperative delirium (OR, 2.35; 95% CI, 1.39–3.96; *p* = 0.0009), with an absolute risk difference of 0.5% (95% CI, 0.2–0.7).

Table 3 presents the results of the multivariable logistic regression analyses performed in the unmatched cohort to identify independent risk factors for postoperative complications that remained significant after PS matching. For deep vein thrombosis, female sex (OR, 1.29; 95% CI, 1.20–1.39; *p* < 0.0001), hypertension (OR, 1.18; 95% CI, 1.12–1.26; *p* < 0.0001), hyperlipidemia (OR, 1.22; 95% CI, 1.14–1.31; *p* < 0.0001), TKA (OR, 1.45; 95% CI, 1.29–1.62; *p* < 0.0001) and simultaneous bilateral surgery (OR, 1.31; 95% CI, 1.17–1.46; *p* < 0.0001) were identified as independent risk factors.

**Table 3:** Multivariate logistic regression analysis of risk factors for deep vein thrombosis and postoperative delirium in the unmatched cohort.

Variable	Deep vein thrombosis		Delirium	
	Odds ratio (95% CI)	*p* value	Odds ratio (95% CI)	*p* value
Age	1.00 (0.99–1.01)	0.31	1.08 (1.06–1.09)	<0.0001*
Women	1.29 (1.20–1.39)	<0.0001*	0.77 (0.81–1.07)	0.022
BMI	0.99 (0.99–1.01)	0.89	0.98 (0.95–1.00)	0.05
CCI	0.93 (0.89–0.97)	0.0007*	1.15 (1.02–1.28)	0.019
Hypertension	1.18 (1.12–1.26)	<0.0001*	1.17 (0.95–1.45)	0.14
Ischaemic cardiac disease	0.84 (0.75–0.95)	0.005	1.16 (0.83–1.61)	0.38
Chronic renal dysfunction	0.95 (0.76–1.19)	0.67	1.14 (0.66–1.99)	0.64
Cerebrovascular disease	1.01 (0.87–1.19)	0.84	1.53 (1.04–2.28)	0.032
Hyperlipidemia	1.22 (1.14–1.31)	<0.0001*	1.13 (0.89–1.44)	0.33
Cognitive impairment	1.41 (1.12–1.78)	0.004	3.82 (2.51–5.82)	<0.0001*
TKA	1.45 (1.29–1.62)	<0.0001*	2.24 (1.42–3.52)	0.0005*
Bilateral surgery	1.31 (1.17–1.46)	<0.0001*	1.25 (0.84–1.86)	0.26

Abbreviations: BMI, body mass index; CI, confidence interval; CCI, Charlson Comorbidity Index; TKA, total knee arthroplasty.

* *p* values of <0.001 are considered significant by the *χ* ^2^ test.

For postoperative delirium, increasing age was independently associated with a higher risk (per 1‐year increase: OR, 1.08; 95% CI, 1.06–1.09; *p* < 0.0001). Cognitive impairment (OR, 3.82; 95% CI, 2.51–5.82; *p* < 0.0001) and TKA (OR, 2.24; 95% CI, 1.42–3.52; *p* = 0.0005) were also identified as independent risk factors.

As an additional analysis, 24,808 matched pairs of patients without Type 2 diabetes mellitus who underwent TKA or UKA were identified after one‐to‐one PS matching. All baseline covariates were well balanced after matching, with SMDs below 0.1 (Supporting Information S1: Table 1). In the matched cohort, significant differences between TKA and UKA were observed in several perioperative outcomes, including deep vein thrombosis, pneumonia, postoperative delirium, length of hospital stay and blood transfusion (Supporting Information S1: Table 2). The ORs for deep vein thrombosis associated with TKA relative to UKA were 1.46 in patients with Type 2 diabetes mellitus and 1.51 in those without diabetes mellitus; the corresponding ORs for postoperative delirium were 2.35 and 1.85, respectively. No significant interaction between diabetes status and surgical procedure was observed for deep vein thrombosis or postoperative delirium (Supporting Information S1: Table 3).

Table 4 summarizes the use of antithrombotic agents in patients undergoing TKA and UKA. Edoxaban was the most frequently prescribed antithrombotic agent in both groups. Before PS matching, patients undergoing TKA had a significantly higher rate of edoxaban use than those undergoing UKA, whereas no significant between‐group differences were observed for the use of other antithrombotic agents. After PS matching, the difference in edoxaban use remained statistically significant, while the use of all other antithrombotic agents remained comparable between the two groups. Overall, patients undergoing TKA were more likely to receive postoperative antithrombotic prophylaxis with edoxaban than those undergoing UKA.

**Table 4:** Comparison of antithrombotic therapies before and after PS matching.

	Before PS matching			After PS matching		
	TKA (*n* = 49373)	UKA (*n* = 5801)	SMD	TKA (*n* = 5783)	UKA (*n* = 5783)	SMD
Edoxaban	29,757 (60.3%)	2829 (48.8%)	0.232	3452 (59.7%)	2820 (48.8%)	0.219
Fondaparinux	976 (2.0%)	149 (2.6%)	0.041	109 (1.9%)	149 (2.6%)	0.047
Enoxaparin	4819 (9.8%)	504 (8.7%)	0.037	546 (9.1%)	501 (8.7%)	0.027
Aspirin	5602 (11.4%)	756 (13.0%)	0.052	651 (11.3%)	753 (13.0%)	0.053
Warfarin	1259 (2.6%)	75 (1.3%)	0.092	130 (2.3%)	75 (1.3%)	0.072
Clopidogrel	1962 (4.0%)	262 (4.5%)	0.027	232 (4.0%)	261 (4.5%)	0.025
Apixaban	1284 (2.6%)	107 (1.8%)	0.051	151 (2.6%)	106 (1.8%)	0.053

*Note*: One‐to‐one PS matching was performed. An SMD < 0.1 was considered indicative of adequate covariate balance.

Abbreviations: PS, propensity score; SMD, standard mean difference; TKA, total knee arthroplasty; UKA, unicompartmental knee arthroplasty.

## DISCUSSION

In this nationwide cohort of patients with Type 2 diabetes undergoing knee arthroplasty, we found that TKA was associated with a higher incidence of deep vein thrombosis and postoperative delirium than UKA, even after meticulous adjustment for demographic factors, comorbidities and anaesthesia type using PS matching. In addition, patients undergoing TKA had longer hospital stays, were less likely to be discharged directly home and received greater transfusion volumes on the day of surgery compared with those undergoing UKA, whereas no significant differences were observed between the two procedures in the incidence of pulmonary embolism, surgical site infection or cerebrovascular events. These findings indicate that, among carefully selected candidates with Type 2 diabetes, UKA may offer a more favourable short‐term perioperative risk profile than TKA, particularly with respect to thromboembolic events, delirium and resource use during hospitalization. Additional analyses in patients without Type 2 diabetes mellitus demonstrated similar differences in perioperative outcomes between TKA and UKA. Furthermore, no significant interactions between diabetes status and surgical procedure were observed for deep vein thrombosis or postoperative delirium, suggesting that these differences were not specific to patients with Type 2 diabetes mellitus.

Previous studies have consistently shown that diabetes is associated with higher rates of periprosthetic joint infection, thromboembolic events, cardiovascular complications and early mortality after TKA, and that patients with diabetes often experience slower recovery of physical function and health‐related quality of life than those without diabetes [3, 10, 40, 44]. In parallel, numerous comparative studies and meta‐analyses have reported that UKA, when performed in appropriately selected patients, is associated with shorter operative time and hospital stay, less blood loss, faster early recovery of knee motion and gait and lower incidences of short‐term complications compared with TKA [16, 30, 41]. Our findings extend this body of evidence by demonstrating that, even within a large cohort restricted to patients with Type 2 diabetes, UKA maintains a more favourable short‐term perioperative profile than TKA, with lower risks of deep vein thrombosis and postoperative delirium and less intensive in‐hospital resource utilization, while the incidence of rare but severe events such as pulmonary embolism and cerebrovascular complications remains comparable between the two procedures.

Several mechanisms may underlie the higher risks of deep vein thrombosis and postoperative delirium and the less favourable in‐hospital course observed after TKA compared with UKA. TKA generally involves more extensive soft tissue dissection, greater bone resection and longer operative time than UKA, which may augment perioperative inflammation, venous stasis in the operated limb and early postoperative immobility [16, 41]. Although diabetes is associated with a prothrombotic state and vascular and neurological abnormalities that may increase susceptibility to perioperative complications [14, 33], our additional analyses showed similar differences between TKA and UKA in patients without Type 2 diabetes mellitus, with no significant interaction between diabetes status and surgical procedure for deep vein thrombosis or postoperative delirium. These findings suggest that the observed differences are more likely attributable primarily to differences in surgical invasiveness between TKA and UKA rather than to a diabetes‐specific effect. Furthermore, the higher transfusion requirements, longer hospital stays and lower rates of direct discharge to home observed after TKA are consistent with the greater physiological burden and slower early recovery associated with the more invasive procedure [32, 46].

From a clinical perspective, our findings suggest that UKA may offer a favourable short‐term perioperative profile for patients with Type 2 diabetes who meet established selection criteria. In appropriately selected individuals with isolated unicompartmental osteoarthritis, UKA may reduce the burden of short‐term complications and facilitate earlier recovery and discharge to home [36, 41]. Nevertheless, UKA is not universally applicable, and many patients with Type 2 diabetes will continue to require TKA because of the extent or pattern of joint disease [36, 41]. For these patients, our findings support careful perioperative risk assessment and appropriate preventive strategies for complications such as deep vein thrombosis and postoperative delirium.

A major strength of this study is the use of a nationwide administrative database combined with PS matching, which enabled effective adjustment for key confounding factors, including age, sex and comorbidities. The substantial sample size further enhances the statistical robustness and precision of our findings. Nevertheless, several limitations should be acknowledged. First, the study population was restricted to patients undergoing knee arthroplasty in acute care hospitals participating in the DPC system. Consequently, individuals admitted to non‐DPC‐reported beds—which represent approximately 30% of all general hospital beds—and those treated outside acute care settings were not included [22]. Second, the accuracy of DPC diagnostic classifications could not be independently validated, nor could the clinical severity of comorbid conditions be assessed at the individual patient level. Third, because postoperative outcomes were defined using ICD‐10 codes from an administrative database, they may encompass a heterogeneous spectrum of clinical conditions, and coding errors or variations in diagnostic practices may have led to misclassification. Moreover, information on diabetes‐related factors such as duration of disease, antidiabetic medication use, insulin therapy and current glycemic control (e.g., HbA1c levels) was not available. Because perioperative complication risks may differ according to the degree of glycemic control and diabetes treatment, heterogeneity in these factors may have influenced the observed outcomes. Therefore, our findings should be interpreted as comparisons between TKA and UKA among patients with a diagnosis of Type 2 diabetes mellitus rather than as an assessment of the effect of glycemic control itself. Fourth, the DPC database does not provide information on institution‐specific postoperative deep vein thrombosis screening protocols. Therefore, we could not determine whether deep vein thrombosis was detected through routine screening or symptom‐triggered evaluation, and differences in screening practices among institutions may have influenced the reported incidence of deep vein thrombosis. Information on mechanical venous thromboembolism prophylaxis and the indications and timing of antithrombotic medication use relative to deep vein thrombosis diagnosis was also unavailable, precluding a reliable assessment of the association between venous thromboembolism prophylaxis and deep vein thrombosis development. Fifth, although most covariates were well balanced after PS matching, unmeasured or incompletely measured confounders—such as radiographic severity and pattern of joint disease, surgeon experience, detailed rehabilitation protocols and chronic use or dosing of antithrombotic agents—may still have influenced both the choice of procedure and postoperative outcomes. Finally, long‐term outcomes such as late infection, periprosthetic fracture, reoperation or post‐discharge mortality were not evaluated, and our findings therefore pertain primarily to short‐term in‐hospital complications.

Despite these limitations, our study has important clinical implications. In a large nationwide cohort of patients with Type 2 diabetes, UKA was associated with lower risks of deep vein thrombosis and postoperative delirium and more favourable in‐hospital recovery profiles than TKA, suggesting that UKA may be a preferable option for appropriately selected diabetic patients undergoing knee arthroplasty. At the same time, many patients with Type 2 diabetes will continue to require TKA, and targeted strategies to optimize perioperative risk factors, strengthen venous thromboembolism prophylaxis and prevent delirium in this high‐risk population remain a priority for future research.

## CONCLUSION

In conclusion, this nationwide PS–matched analysis demonstrated that, among patients with Type 2 diabetes undergoing knee arthroplasty, TKA was associated with higher risks of deep vein thrombosis and postoperative delirium, longer hospital stays, lower rates of direct discharge to home and greater transfusion requirements than UKA, while rates of pulmonary embolism, surgical site infection and cerebrovascular events were comparable between the two procedures. Similar differences were observed in patients without Type 2 diabetes, with no significant interaction between the presence of Type 2 diabetes mellitus and surgical procedure for deep vein thrombosis or postoperative delirium. These findings suggest that the differences in short‐term perioperative outcomes between TKA and UKA are not specific to diabetes, while confirming that UKA has a more favourable short‐term perioperative profile than TKA among appropriately selected patients with Type 2 diabetes.

## AUTHOR CONTRIBUTIONS

Yu Mori and Hidetatsu Tanaka conceived and designed the study. Yu Mori performed the statistical analyses and drafted the manuscript. Kunio Tarasawa, Ryuichi Kanabuchi and Naoko Mori contributed to data interpretation. Kiyohide Fushimi and Kenji Fujimori contributed to data acquisition and database management. Toshimi Aizawa supervised the study. All authors critically revised the manuscript and approved the final version.

## FUNDING INFORMATION

The authors have no funding to report.

## CONFLICT OF INTEREST STATEMENT

The authors declare no conflicts of interest.

## ETHICS STATEMENT

This study was conducted in accordance with the Declaration of Helsinki and was approved by the Institutional Review Board of Tohoku University (approval number: 2024‐1‐1026).

## Supporting information

Supplementary Tables_20260831.

## Data Availability

The data that support the findings of this study are available from the Japanese Diagnosis Procedure Combination (DPC) database. Restrictions apply to the availability of these data, which were used under license for the current study, and so are not publicly available.
