# Supplementary material for: Perioperative outcomes of total and unicompartmental knee arthroplasty in patients with Type 2 diabetes mellitus: A propensity score–matched analysis
Source: J Exp Orthop. 2026 Sep 22;13(3):e70917. doi: 10.1002/jeo2.70917 (PMC13596008; doi:10.1002/jeo2.70917)
Supplement: Supplementary file 1 [file JEO2-13-e70917-s001.docx]

Supplementary Table 1. Characteristics of patients without type 2 diabetes mellitus before and after propensity score matching

|  | Before PS matching | | | After PS matching | | |
| --- | --- | --- | --- | --- | --- | --- |
|  | TKA | UKA | SMD | TKA | UKA | SMD |
| n | 179222 | 24923 |  | 24808 | 24808 |  |
| Sex |  |  |  |  |  |  |
| Men | 36245 (20.2%) | 6475 (26.0%) | 0.137 | 6346 (25.6%) | 6453 (26.0%) | 0.010 |
| Woman | 142977 (79.8%) | 18448 (74.0%) |  | 18462 (74.4%) | 18355 (74.0%) |  |
| Age | 74.8 ± 7.9 | 74.3 ± 7.7 | 0.064 | 74.4 ± 8.0 | 74.3± 7.7 | 0.013 |
| BMI | 25.9 ± 4.8 | 25.1 ± 4.3 | 0.176 | 25.0 ± 3.9 | 25.0 ± 3.5 | 0.009 |
| CCI | 0.47 ± 0.83 | 0.33 ± 0.72 | 0.180 | 0.32 ± 0.68 | 0.33 ± 0.72 | 0.014 |
| Hypertension | 61348 (34.3%) | 7737 (31.0%) | 0.068 | 7500 (30.2%) | 7711 (31.1%) | 0.018 |
| Ischemic cardiac disease | 9713 (5.4%) | 1229 (4.9%) | 0.022 | 1155 (4.7%) | 1222 (4.9%) | 0.013 |
| Chronic renal dysfunction | 5150 (2.9%) | 576 (2.3%) | 0.035 | 519 (2.1%) | 574 (2.3%) | 0.015 |
| Cerebrovascular disease | 5504 (3.1%) | 651 (2.6%) | 0.028 | 583 (2.4%) | 650 (2.6%) | 0.017 |
| Hyperlipidemia | 34181 (19.1%) | 4774 (19.2%) | 0.002 | 4506 (18.2%) | 4744 (19.1%) | 0.025 |
| Cognitive impairment | 2930 (1.6%) | 318 (1.3%) | 0.030 | 297 (1.2%) | 317 (1.3%) | 0.007 |
| General anesthesia | 163327 (91.1%) | 21805 (87.5%) | 0.118 | 21860 (88.1%) | 21719 (87.6%) | 0.017 |
| Bilateral surgery | 12132 (6.8%) | 1042 (4.2%) | 0.114 | 1016 (4.1%) | 1041 (4.2%) | 0.005 |

One-to-one PS matching was performed.

Data is shown as mean ± standard deviation.

An SMD < 0.1 was considered indicative of adequate covariate balance.

PS means propensity score; SMD means standard mean difference; BMI means body mass index; CCI means Charlson comorbidity index; TKA means total knee arthroplasty; UKA means unicompartmental knee arthroplasty.

Supplementary Table 2. Comparison of complications in patients without type 2 diabetes mellitus before and after propensity score matching

|  | Before PS matching | | | After PS matching | | | |
| --- | --- | --- | --- | --- | --- | --- | --- |
|  | TKA  (n=179222) | UKA  (n=24923) | *p*-value | TKA  (n=24808) | UKA  (n=24808) | Odds ratio | *p*-value |
|  |  |  |  |  |  | 95% CI |  |
| Deep vein thrombosis | 16785 (9.4%) | 1521 (6.1%) | < 0.0001* | 2294 (9.3%) | 1515 (6.1%) | 1.51  (1.42-1.61) | < 0.0001* |
| Pulmonary embolism | 530 (0.3%) | 37 (0.2%) | < 0.0001* | 65 (0.3%) | 37 (0.1%) | 1.76  (1.17-2.63) | 0.0055 |
| Pneumonia | 345 (0.2%) | 24 (0.1%) | 0.0008* | 52 (0.2%) | 23 (0.1%) | 2.26  (1.38-3.70) | 0.0008* |
| Delirium | 1341 (0.8%) | 103 (0.4%) | < 0.0001* | 189 (0.8%) | 102 (0.4%) | 1.85  (1.46-2.36) | < 0.0001* |
| Cerebrovascular event | 548 (0.3%) | 45 (0.2%) | 0.0006* | 60 (0.2%) | 45 (0.2%) | 1.33  (0.91-1.96) | 0.14 |
| Surgical site infection | 2123 (1.2%) | 190 (0.8%) | < 0.0001* | 259 (1.0%) | 190 (0.8%) | 1.36  (1.13-1.64) | 0.0011 |
| Periprosthetic fracture | 111 (0.1%) | 38 (0.2%) | < 0.0001* | 15 (0.1%) | 38 (0.2%) | 0.39  (0.22-0.72) | 0.0016 |
| Length of hospitalization (days) | 28.4 ± 16.0 | 22.7 ± 11.1 | < 0.0001* | 28.0 ± 21.1 | 22.7 ± 11.1 |  | < 0.0001* |
| Discharge to home | 148883 (83.1%) | 22958 (92.1%) | < 0.0001* | 20770 (83.7%) | 22852 (92.1%) | 0.91  (0.90-0.91) | < 0.0001* |
| Blood transfusion Day 0 (unit) | 0.06 ± 0.42 | 0.01 ± 0.12 | < 0.0001* | 0.06 ± 0.41 | 0.01 ± 0.12 |  | < 0.0001* |
| One-to-one PS matching was performed. Odds ratios are expressed for TKA relative to UKA (reference). | | | | | | | |
| **p*-values of < 0.001 are considered significant by the χ^2^ test and Student’s t-test.  PS means propensity score; CI means confidence interval; TKA means total knee arthroplasty; UKA means unicompartmental knee arthroplasty. | | | | | | | |

Supplementary Table 3. Interaction between diabetes status and surgical procedure for postoperative complications

|  | Type 2 DM TKA vs UKA OR | non-DM TKA vs UKA OR | *p-*value for interaction |
| --- | --- | --- | --- |
| Deep vein thrombosis | 1.46 | 1.51 | 0.27 |
| Delirium | 2.35 | 1.85 | 0.33 |

One-to-one PS matching was performed separately in patients with and without type 2 diabetes mellitus. OR are expressed for TKA relative to UKA (reference) in the PS-matched cohorts. The interaction between diabetes status and surgical procedure was assessed using the overall cohort before PS matching. The *p-*value for interaction represents the interaction between diabetes status and surgical procedure.

PS means propensity score; OR means odds ratio; TKA means total knee arthroplasty; UKA means unicompartmental knee arthroplasty; DM means diabetes mellitus.
